# Body mass and cancer: genetics, endocrinology… and more

**DOI:** 10.4155/fsoa-2016-0090

**Published:** 2017-01-20

**Authors:** Lev M Berstein

**Affiliations:** 1 Laboratory of Oncoendocrinology, N.N. Petrov Research Institute of Oncology, Pesochny-2, St Petersburg 197758, Russia

**Keywords:** BMI, breast cancer, endometrial cancer, genetic score, obesity phenotypes, pre- and post-menopausal

An article by Guo *et al.* [1] published in August 2016 should interest any researcher involved in the study of the connection between body mass values and cancer incidence, which is especially evident for breast cancer. The traditional and seemingly established point of view, which originates, at least, from the research of de Waard [2], considers women of reproductive age with higher BMI to be less inclined to breast cancer development compared with women with normal BMI values, although the situation reverses after the menopause (i.e., becomes high BMI/high breast cancer risk). These differences are usually explained (and by Guo *et al.* [1]) by an ‘estrogen factor’ role. In particular, in the first (premenopausal) group, the relationship is explained by the influence of anovulatory menstrual cycles, less estrogen production and consequent breast epithelium proliferation signal attenuation. In the second (post-menopausal) group, the excess of adipose tissue derived estrogens, which leads to their higher mean serum level compared with women with normal body mass, is considered the most important related risk factor.

In the article under discussion here, the authors drifted away from the usual epidemiological approach to the Mendelian randomization method, allowing determination of the cause–effect relations between potential risk factors and disease based on genetic data analysis. For this reason, they established a BMI genetic score that included 84 BMI-associated genetic variants. The data for the study were received from two major databases – Breast Cancer Association Consortium and Discovery, Biology, and Risk of Inherited Variants in Breast Cancer – with a total of approximately 62,000 entries on breast cancer patients and 83,000 entries on healthy women. For both databases, a genetically predicted BMI had a highly significant inverse correlation to breast cancer risk, which was evident for both pre- and post-menopausal groups, in direct contradiction to ‘common’ conceptions. Thus, based on the data, the breast cancer risk (OR) for BMI >26.6 versus <25.0 was equal for pre- and post-menopausal females at 0.78 and 0.88, respectively, while for progressive BMI increase (with 5 kg/m^2^ steps), the OR for those groups appeared to be 0.44 and 0.57, respectively [1]. Individually, 17 of 84 studied BMI-associated SNPs were related to breast cancer risk, and, in fact, in 16 of these 17 cases the allele associated with elevated BMI connected with reduced breast cancer risk.

While the authors realize the unconventionality of the results obtained, they have come to the preliminary conclusion that genetic prediction of BMI/breast cancer relation is more likely to be important for the early or midlife period, while high BMI values may be influenced later in life by environmental factors associated with an increased risk of breast cancer [1].

As is well known, dogmas are often persistent, although in some cases they gradually relax their grip and give way to other ideas and principles. Will it happen in this case?

To make a ‘prediction’ we must discuss several points that deserve attention.

First of all, the study by Guo *et al.* [1] was performed in a cohort of European descent; we cannot say now whether or not the described relationships remain true in Asian or African populations for there are known racial and ethnic discrepancies in the role of BMI as a potential breast cancer risk modifier [3].

Second, an excessive BMI value as a cancer risk factor is not equivalent to obesity [4], which implies a need for body composition (in particular fat-to-lean body mass ratio) analysis. This same point is even more important considering obesity heterogeneity [5], and taking into account the possibility of obesity division into ‘standard’ (with insulin resistance-type metabolic disorders) and ‘metabolically healthy’ (without these changes) categories [6].

Third, the BMI value cannot apparently be viewed in the context of breast canсer risk in females of different ages without considering the notion of fitness, the role of which was demonstrated in studies of the relation of BMI to cardiovascular pathology [7]. On the other hand, the connection between physical activity and breast cancer risk can also be modified by genetic factors [8].

Fourth, aside from factors seemingly distanced from the breast epithelium as a target tissue and mentioned by Guo *et al.* [1], such as central mechanisms of BMI regulation [9], we must not forget the local modifiers of breast cancer risk and progression. In particular, in the post-menopausal period the breast cancer risk is determined not only by serum estrogen levels (substantially dependent on their adipose tissue production), but in no small part by estrogens synthesized in breast tissue itself. Unlike ovarian estrogen synthesis, this local production is not diminished with age [10] and, therefore, must be accounted for in comparison of pre- and post-menopausal cases.

Fifth, according to a recent observation, the Mendelian randomization method allowed establishment of a significant positive correlation between BMI-associated genetic risk score and endometrial cancer risk in a female cohort of mostly post-menopausal age (as opposed to breast cancer, where the association was negative). Of note, the correlation in this case was even more significant than the one for BMI itself [11]. Whether related to tissue specificity of these two cancers, the different actions of hormone-associated factors or cancer subtype diversity [12–14] are undoubtedly important matters for future research and analysis.

Finally, the question of whether one should review (based on the genetic analysis data of Guo *et al.* [1]) existing concepts on need for weight loss intervention and avoidance of weight gain in breast cancer [15] remains thus far open.

In conclusion, we should acknowledge that the unexpected results of the study by Guo *et al.* [1] imply a need for further research of hormone-dependent tissue tumors as these results have a fundamental as well as applied relevance.
